# Determination of the center of mass in a heterogeneous population of dogs

**DOI:** 10.1371/journal.pone.0267361

**Published:** 2022-04-27

**Authors:** Tiffany A. Johnson, Wanda J. Gordon-Evans, B. Duncan X. Lascelles, Michael G. Conzemius

**Affiliations:** 1 Department of Veterinary Clinical Sciences, University of Minnesota, College of Veterinary Medicine, Saint Paul, Minnesota, United States of America; 2 Department of Clinical Sciences, North Carolina State University College of Veterinary Medicine, Translational Research in Pain, Comparative Pain Research and Education Center, Raleigh, North Carolina, United States of America; Thomas Jefferson University, UNITED STATES

## Abstract

The center of mass (CoM) is the location in a body where mass distribution is balanced. It has a fundamental role in balance and motion which has been poorly described in the dog. The objective of this study was to estimate the variance of the center of mass (CoM) in a heterogeneous population of client-owned dogs and to describe the relationship between CoM, subject morphometrics and an inertial measurement unit (IMU) box positioned ventrally on a neck collar. A single force platform and a reaction board were used to determine CoM in the transverse, sagittal and dorsal planes in thirty-one healthy adult dogs. A series of morphometric measurements were acquired with each dog standing, including distances relative to an IMU box positioned ventrally on a neck collar. Mean transverse plane CoM was 48% the distance from ischium to the IMU box, near the xiphoid process. Mean sagittal place CoM was 59% the width of the chest on the left side. Mean dorsal plane CoM was 41% the distance from the most dorsal to the most ventral aspect of the body. Dog length was the primary variable required to maximize the relationship between three-dimensional CoM and identifiable variables measured. A CoM based normalization procedure should be considered to normalize mass or motion based outcome measure output (e.g., ground reaction forces, vector acceleration) in a heterogeneous population of dogs. Future research will be needed to determine if CoM-based normalization procedures reduce variance in outcome measures affected by subject morphometrics.

## Introduction

Translational research using spontaneous occurring models of osteoarthritis (OA) [1–3] and pain [4] in the dog can be limited when objective outcome measures are used in a heterogeneous population [5–7]. The size and shape variability in dogs impacts ground reaction forces [7,8] and accelerometer output; [6] commonly used outcome measures in studies of spontaneous models of canine OA and chronic pain. Understanding the relationship between the subject center of mass (CoM) and morphometrics (e.g., weight, height, length) in a heterogeneous population of dogs may help guide future investigations using these outcome measures.

The CoM of a dog can be described as the location in the body where the distribution of mass is balanced. It can also be described as the unique point in planes dividing the body into two parts [9]. Controlling and propelling CoM is important in locomotion, balance and movement [10,11]. During motion, there are constant changes in the CoM because of positional changes in the body and, like the human body [11], the canine body has a complicated shape and is built from many tissues of different densities. Thus, to help put the CoM in context, it can be described as a distance from an anatomic reference or a reference system [11,12].

The objectives of this study were to identify the variance of CoM in the transverse, sagittal and dorsal planes in a heterogeneous population of client-owned dogs and describe the relationship between three-dimensional CoM, subject morphometrics and an inertial measurement unit (IMU) box positioned ventrally on a neck collar. We studied the hypothesis that three-dimensional subject CoM would be influenced by subject morphometrics.

## Materials and methods

Institutional Animal Care and Use Committee approval (2103-38908A) and written, informed client consent was required for patient inclusion. Owners received compensation for participation in the study, which they were made aware of before enrollment in the study. The owner report and physical examination performed before entry into the study determined subject’s health status. Healthy adult dogs that were not pregnant were recruited for this study.

A single force platform (OR6 6 1000, Advanced Mechanical Technology Inc, Watertown, MA, 02472) was used in the study and validated before each use via a preexisting standard operating procedure using dedicated software (Sharon Software, Inc. Dewitt, MI, 48820) that included demonstrating a homogenous, cast iron, rectangular 25.0kg grip handle test weight certified by the International Organization of Legal Metrology (International Bureau of Legal Metrology, 11, rue Turgot, 75009 Paris, France) reported as 25.0kg. A reaction board [12–16] is a platform that has one end rest on a force platform and is used to support the subject. It has a known CoM that is accounted for in calculating the subject’s CoM. The reaction board was made of pinewood and three, 1 mm steel L-braces that were each secured to the board with six steel screws. The dimensions of the board were measured and it was weighed. For the purposes of this study the reaction board was considered to have uniform density. Calibration of the reaction board was done by moving the 25.0kg grip handle test weight along the length board covering the range of subject locations on the reaction board to evaluate known CoM to measured CoM [12].

Data recorded from dogs enrolled in the study included breed, age, gender, body condition score, and body weight. A neck collar was placed on each dog by a single investigator so two fingers could fit under the collar. The collar held a wearable IMU box that was positioned ventrally on the dog’s neck and a series of measurements were acquired with the dog standing: 1) IMU box to ground, 2) IMU box to ischium, 3) ventral part of chest to ground (allowing for calculation: IMU box to ground–ventral part of chest to ground = IMU box to most ventral part of chest), 4) IMU box to acromion, 5) height to right and left acromion, 6) distance from acromion to acromion, 7) height to right and left greater trochanter, 8) distance between trochanters, 9) distance from acromion to trochanter on the left and right, 10) right and left front foot to right and left rear foot (measured back of foot to back of foot), and 11) right and left inter-foot distance (measured from inside of foot to inside of foot). Measurements were taken by the same investigator using a tape measure or a large caliper.

Dogs were lightly sedated, to effect, using intravenous dexmedetomidine (1.0–4.0 mcg/kg) and butorphanol (0.1–0.4 mg/kg) so they could be properly positioned and remain motionless on the reaction board during CoM measurements. Following CoM measurements, sedation was reversed using Atipamezole (an equal volume of dexmedetomidine), and dogs were observed until they could easily ambulate without assistance and then returned to their owners.

**Fig 1:**
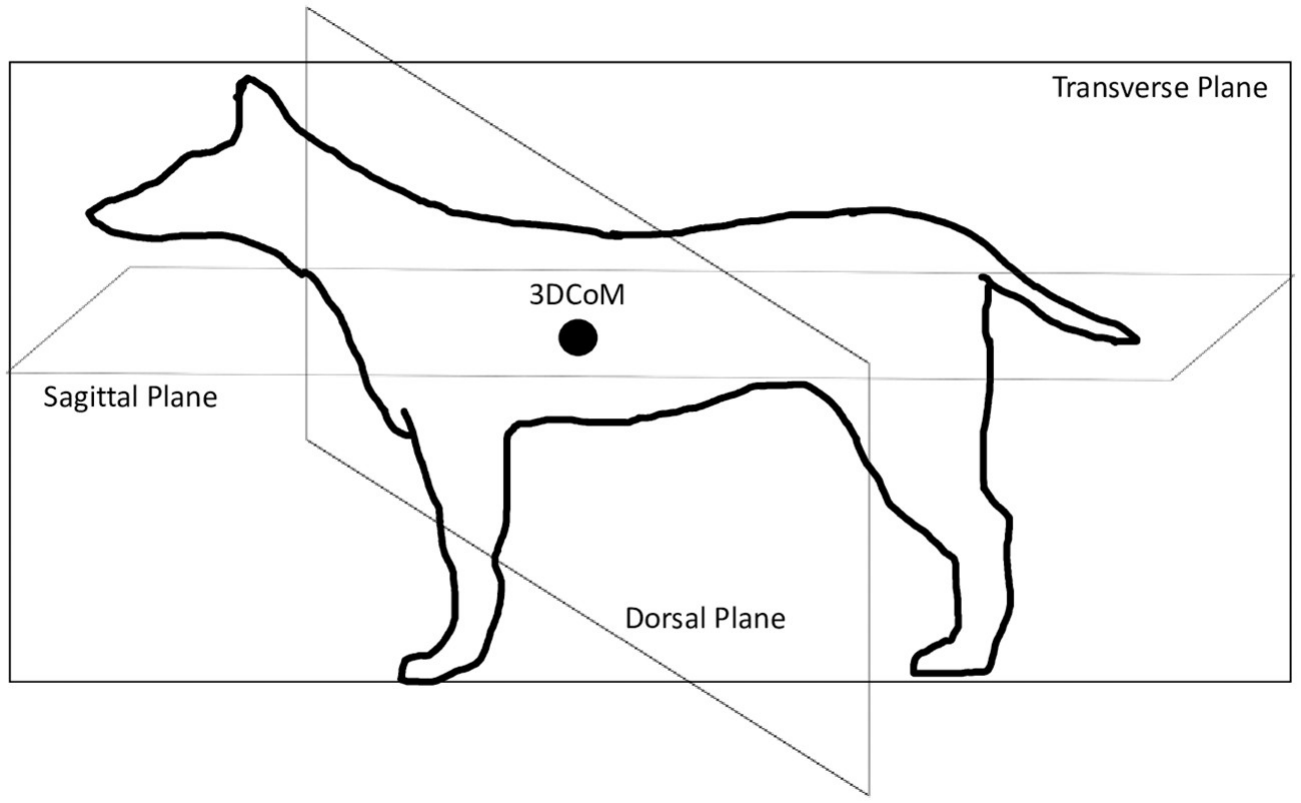
Illustration of the transverse, sagittal and dorsal planes of a dog with a fictitious 3DCoM (black dot).

**Fig 2:**
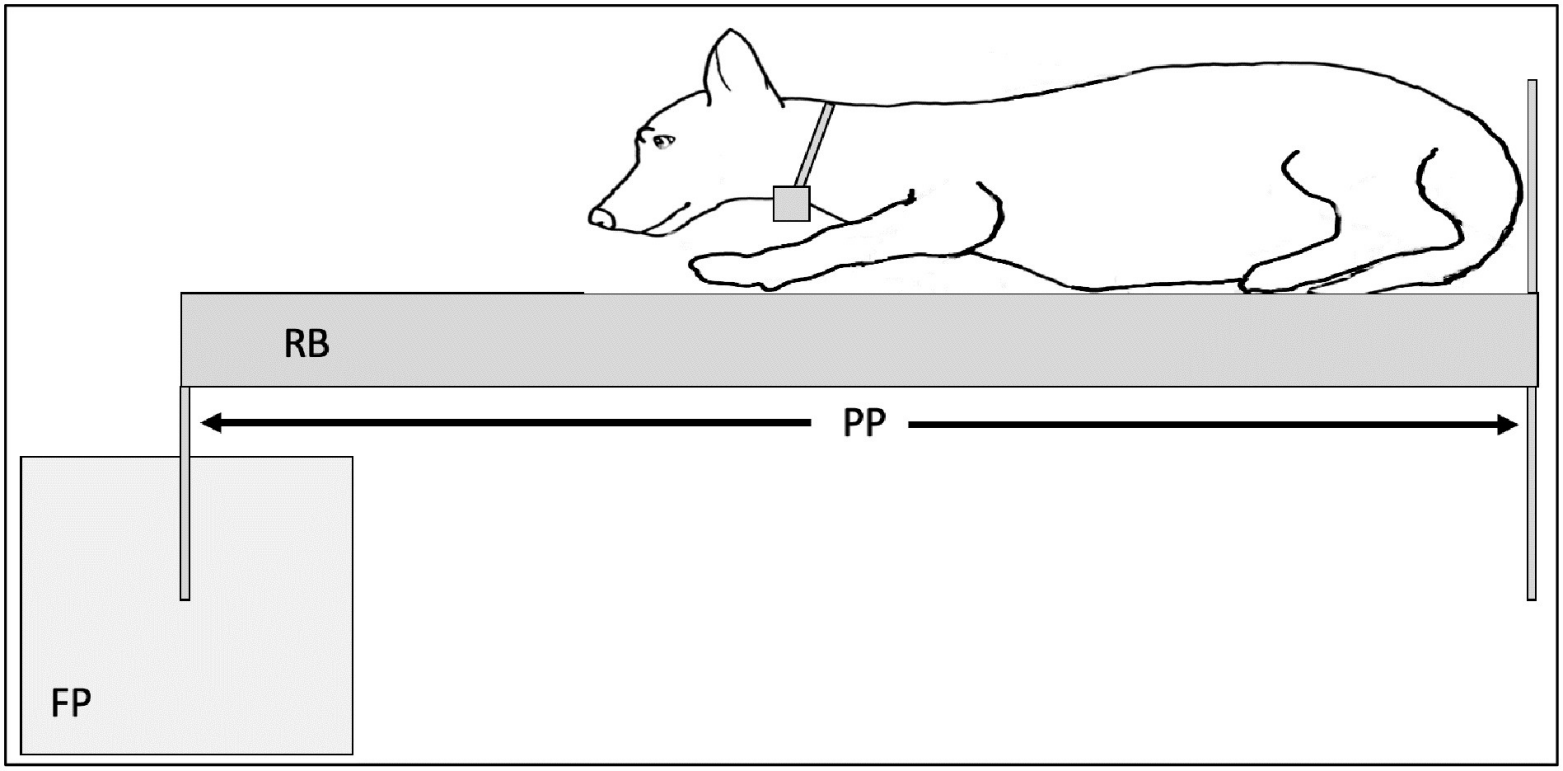
Illustration of a dog positioned on the reaction board (RB) for the measurement of center of mass in the dog’s transverse plane. The dog’s ischium is in contact with the back of the reaction board and the pivot points (PP) are in contact with the floor and force platform (FP).

The CoM can be defined by each plane (transverse, sagittal and dorsal) and a three-dimensional CoM (Fig 1). Center of mass in each dog’s transverse, sagittal and dorsal plane was calculated using measurements from each plane, a reaction board and a force platform. To measure transverse plane CoM (rostral to caudal; nose to ischium), dogs were placed on the reaction board lying in sternal recumbency (tape was used if needed to maintain positioning) with their tail underneath their body, their legs were folded under their body by flexing their shoulder and elbow (front limb) and hip, knee and hock (rear limb) and their ischium was placed adjacent to the reaction board pivot axis (Fig 2). Once positioned and measured, ground reaction force (GRF) was measured by the force platform and transverse plane CoM was calculated: transverse plane CoM = (reaction board length (cm) x transverse plane GRF(N))/dog mass(N). For sagittal plane CoM (right to left; width of chest), dogs were placed on the board lying in sternal recumbency with their tail underneath their body and their legs folded under their body by flexing their shoulder and elbow (front limb) and hip, knee and hock (rear limb) and their right side adjacent to the pivot axis. Once positioned and measured, GRF was measured and sagittal plane CoM was calculated: sagittal plane CoM = (reaction board length (cm) x sagittal plane GRF(N))/dog mass(N). For dorsal plane CoM (dorsal to ventral; withers to ventral part of chest), dogs were placed on the board lying in right lateral recumbency with their head, back and pelvis adjacent to the reaction board pivot axis. Once positioned and measured, GFR was measured and dorsal plane CoM was calculated: CoM dorsal plane = (reaction board length (cm) x dorsal plane GRF(N))/dog mass(N). To calculate the distance from the three dimensional or whole-body CoM (3DCoM) [12] (the dog’s CoM calculated from the transverse, dorsal and sagittal planes) to the IMU box the following steps were taken:

1. Subtract transverse plane CoM from the IMU box to ischium distance.
2. Subtract dorsal plane CoM from the measured dorsal plane distance and subtract this result from IMU box to ventral body (sternum) distance.
3. Utilize Pythagorean theorem to calculate the distance from the transverse-dorsal CoM to the IMU = SQRT((#1)^2^ +(#2)^2^).
4. Subtract sagittal plane CoM from the measured sagittal width/2.
5. Utilize Pythagorean theorem to calculate the distance from the three-dimensional CoM to the IMU box = SQRT((#3)^2^ +(#4)^2^).

### Statistical analysis

No previously published data was found to guide study power, so after 15 dogs completed the study descriptive statistics (including sample variance), correlations between CoM and commonly measured parameters, confidence intervals and power analysis (alpha = 0.05, Power 80%) were performed to help guide the number of animals that needed to be studied. Following initial statistical analysis, it was determined that 31 dogs were required to limit statistical error.

The statistical analysis (R version (2020) 4.0.3 R Core Team) was performed in three parts. First, means, standard deviations, medians and histograms were used to assess each variable for spurious observations. Second, an all-subsets regression procedure (the "regsubsets" function in R and the Bayesian Information Criterion) was used to find a parsimonious regression model that maximized the r^2^ between the predictors and three-dimensional CoM. Third, the fit of the linear regression model resulting from the all-subsets regression was assessed for fit by checking the normality of the residuals with a normal quantile plot and the Shapiro-Wilk test and by a scatterplot of the predicted values and three-dimensional CoM. For simplicity, any predictor variables without statistically significant coefficients were removed from the model, and the model’s fit rechecked as described above.

## Results

Thirty-one healthy adult dogs were enrolled and completed the study. The population included 15 spayed females, one intact female, 12 neutered males and 3 intact male dogs. The mean subject age was 6.23 years (SD: 3.19 years; range: 1.5 to 12 years). Mean subject body weight was 25.58kg (SD: 13.22 kg; range: 6.5 to 60kg).

**Fig 3:**
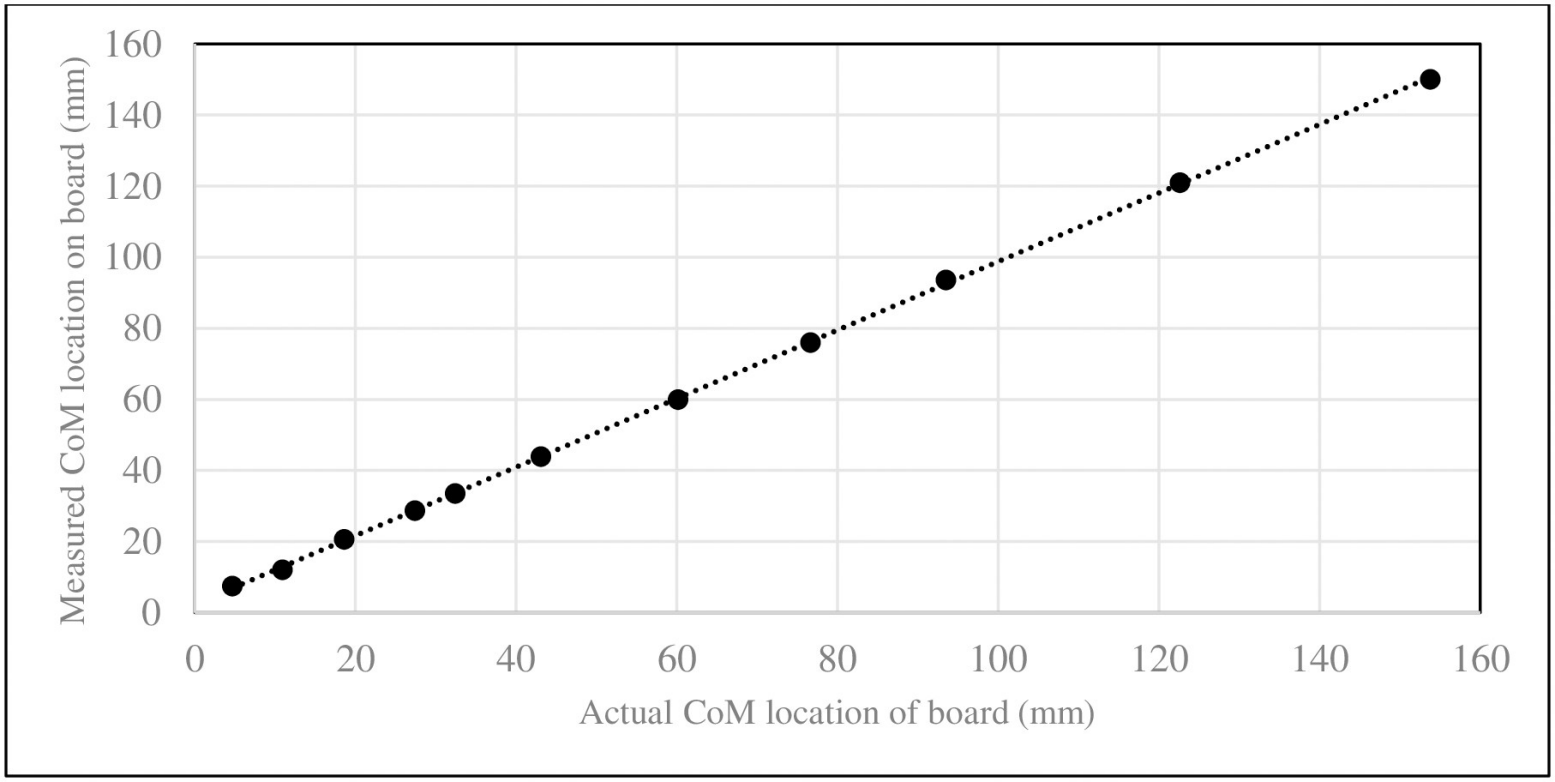
Calibration of the reaction board comparing the difference between the actual position of CoM of the calibrated 25-kg mass and the position measured by the reaction board.

Calibration of the reaction board identified a mean difference between the true position of CoM of the calibrated 25kg mass and the position measured by the reaction board was 0.27mm (SD: 1.84mm; range: -2.80 to 3.80 mm). The coefficient of determination between actual and measured CoM location along the board was R^2^ = 0.9999 (Fig 3).

The mean (±SD) transverse plane length (nose to ischium) was 86.48±20.68cm. The mean (±SD) distance from the IMU box to the ischium was 64.16±15.66cm. The mean (±SD) distance from the transverse plane CoM to the ischium was 33.55±7.22cm. The mean (±SD) sagittal plane width (width of the chest) was 18.07±4.23cm. The mean (±SD) distance from the sagittal plane CoM to the right side of the dog was 10.65±3.61cm. The mean (±SD) dorsal plane height (withers to the ventral part of the chest) was 23.4±5.59cm. The mean (±SD) distance from the withers to the dorsal plane CoM was 9.48±4.44cm. The mean (±SD) distance from the 3DCoM to the IMU box was 33.23±13.47cm.

**Fig 4:**
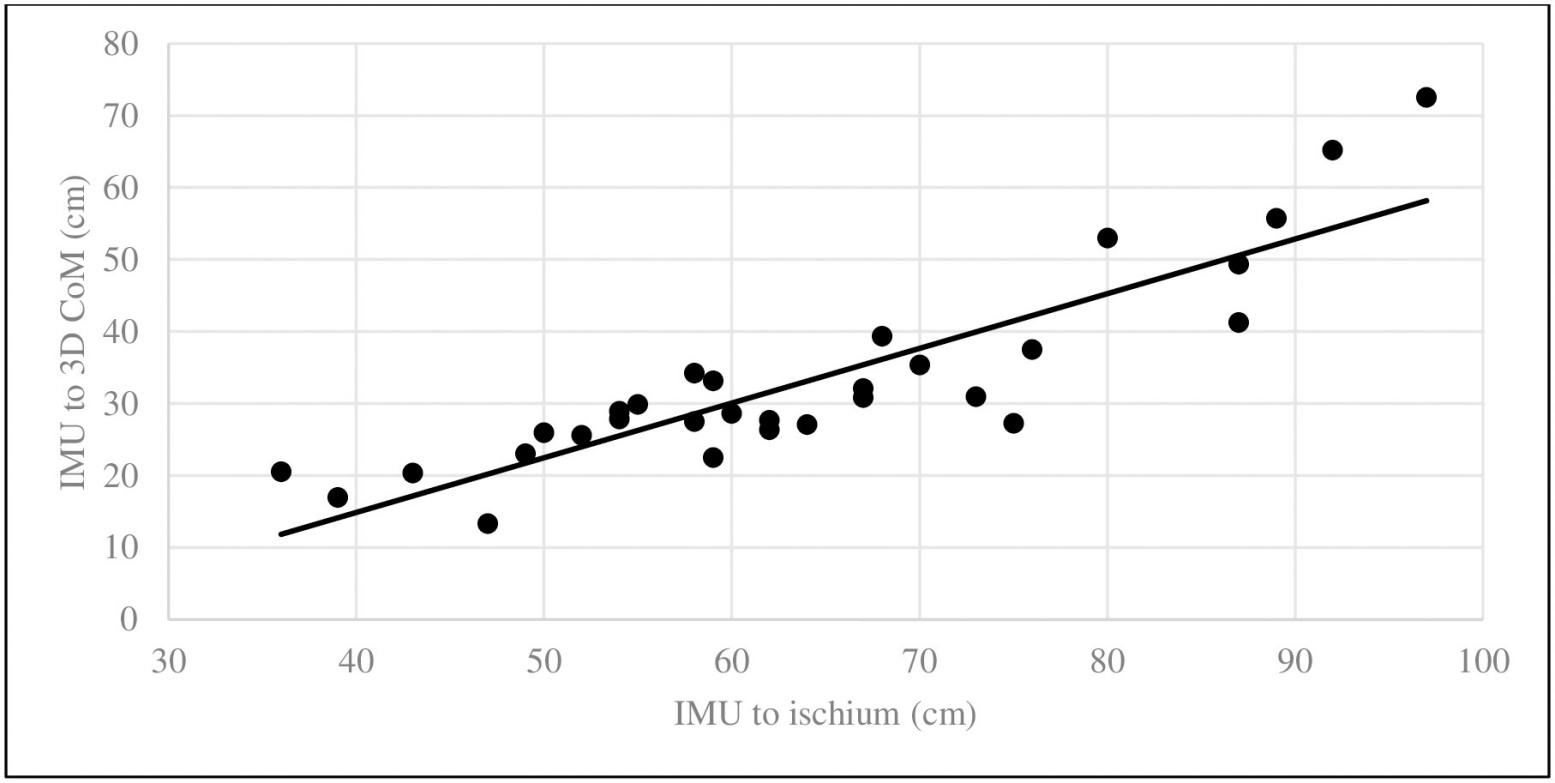
Linear regression between IMU location to subject ischium distance and IMU location to subject 3DCoM. This relationship had a R^2^ = 0.78, p = 4.85^−11^ with normality of residuals (p = 0.91).

With respect to describing the relationship between the 3DCoM, morphometric variables and IMU box, an all-subsets regression identified distance from the IMU box to ischium as the variable that maximized r^2^ without overfitting the data. The simple linear regression of IMU to CoM and IMU to ischium had an r^2^ = 0.78 and p = 4.85^−11^. (Fig 4) These residuals were normally distributed (p = 0.91). The formula, 3DCoM to IMU box = -15.50713 + (0.75962*IMU box to ischium), could be used to normalize distance from the IMU box to the CoM for the dogs in this study.

## Discussion

In this population of dogs, the standard deviation of the mean distance from the 3DCoM to the IMU box had an 81% dispersion. This wide distribution allowed us to accept our hypothesis that subject 3DCoM would be influenced by subject morphometrics. Our finding that dog length was the only variable required to maximize r^2^, parallels findings in people where subject height is the primary physical characteristic to estimate CoM [12,17]. To provide anatomical context to CoM in dogs, we found the transverse plane CoM to be approximately 48% of the distance from the IMU box (ventral part of the neck) to the ischium. Although the CoM changes during motion and there is individual variation, the transverse plane CoM would be in the region of the xiphoid process in dogs. Sagittal plane CoM was just on the left side of midline and dorsal plane CoM was approximately 40% of the distance from the most dorsal to the most ventral aspect of the dogs. It is important to note that subject body weight is implicitly a component of CoM since it is the dividend in the CoM equation used. In contrast to people, we did not find that gender influenced CoM location in this population of dogs [12,18].

The reaction board was constructed, and force platform used, in a manner similar to previous descriptions [12–16]. This included minimizing contact of the reaction board with the ground and force platform by using braces with a 1-mm diameter width. Using a validated 25kg mass, calibration of the reaction board found an average difference between the true and estimated position of the CoM to be 0.27 ± 1.84mm, thus providing a reliable method for determining the reference values in study subjects.

The equation to normalize distance from the IMU box to the CoM for the dogs in this study would not allow one to identify the CoM of a dog; it could reduce variability in CoM differences in a population of dogs. This may be helpful in heterogeneous populations are studied and the outcome measures could be influenced by CoM (considers both subject’s mass and morphometrics). Ground reaction forces (GRFs) in dogs are commonly normalized to address differences in subject morphometrics (e.g. body weight) so the forces generated by a heterogeneous population of dogs can be compared [7]. Normalization of GRFs to body size, using withers height, has also been reported to provide an additional reduction in the coefficient of variation. [7,19] While we are unaware of research exploring this concept, normalization to subject CoM may further reduce variance when measuring GRF in a heterogeneous population of dogs.

We elected to use an IMU box on the ventral part of the neck of the dog as a point of reference because it is a common place for activity monitors to be placed in clinical trials [20–23]. Inertial measurement units are motion-based sensors that offer an opportunity to monitor the activity of a canine patient in their natural environment; information that would be useful for determining the impact of disease burden and treatments for osteoarthritis, chronic pain, cardiovascular disease, and obesity. While many IMUs are commercially available (i.e., activity monitors) for use in dogs, the authors are unaware of validated algorithms for dogs. A direct translation of human IMU algorithms are unlikely to be accurate in dogs, given that dogs are quadrupeds and they have profound variability in size and shape (e.g., Chihuahua to Great Dane). One goal of an IMU algorithm is to improve the reproducibility of IMU output between study subjects; this is particularly important as heterogeneity in the study population increases. The location of the IMU on the subject is important because a location change can affect what the IMU reports. An IMU worn on the wrist or waist of a person results in different reports of the level of activity [24–27]. IMUs worn on the waist are nearer the CoM of a person (navel) and have been shown to be a more accurate representation of energy used during activity [28–30]. However, IMUs are generally worn on the wrist to improve user compliance [27,31]. The difference in acceleration output from an IMU is influenced by distance from the CoM because the CoM is the single location where force can induce linear acceleration without angular acceleration. Additional research will be required to determine if the normalization to subject CoM reduces variance in IMU output in a heterogeneous population of dogs performing similar activities of various intensities.

Beyond normalization procedures for outcome measures, CoM is important in locomotion, balance and movement [10,11]. In this context, change in CoM after amputation or after application of an external prosthetic should be understood to assist in the stabilization and propulsion of the patient [32]. Similarly, helping patients with neurologic disease control, their CoM has been described [33].

There are several limitations to this study. First, this is an estimate of CoM because dogs, and their organs, are in constant motion; they are not rigid bodies nor do all their tissues have a uniform density. Second, the tail and legs were tucked under each dog’s body to reduce measurement error. This leads to a generalization of body segment CoM measurements in the transverse, sagittal and dorsal planes and eliminates the possibility of calculating body segment CoM. In a previous study in people using similar methods [14], investigators first measured CoM with the subject’s arms by their side while standing and lying down. The methods were repeated with the arms raised to calculate the CoM of the forearm body segments. However, they assumed that the subject’s body would be symmetrical in the sagittal plane [14]. While we did not calculate the CoM of body segments, we did not assume that dogs would have left to right symmetry. This allowed for a more precise calculation of the distance from the 3DCoM to the IMU box and other landmarks; that said, the investigation of sagittal plane asymmetry only changed the distance from the CoM to the IMU box by 0.31±0.07cm. Finally, measurement error was not calculated. To limit measurement error, all measurements were taken by a single investigator using the same technique and instruments.

## Conclusions

The methods described in this study determined subject-specific CoM locations in dogs of different morphology. The CoM in this heterogeneous population of dogs was near the xiphoid process in the transverse plane, just to the left side of the midline in the sagittal plane, and 40% of the distance from the most dorsal to the most ventral part of the chest in the dorsal plane. The relationship between the 3DCoM and subject landmarks measured identified distance from the IMU box to ischium as the variable that maximized R^2^. Additional research will be needed to determine if these findings are clinically significant and can be used as a normalization procedure to reduce variability in ground reaction force or IMU output in a heterogeneous population of dogs.

## Supporting information

S1 Data (XLSX)

## References

[1] Vainio O. Translational animal models using veterinary patients—An example of canine osteoarthritis (OA). Scand J Pain. 2012 Apr 1;3(2):84–89. doi: 10.1016/j.sjpain.2011.11.007 . 29913782

[2] Meeson RL, Todhunter RJ, Blunn G, Nuki G, Pitsillides AA. Spontaneous dog osteoarthritis—a One Medicine vision. Nat Rev Rheumatol. 2019 May;15(5):273–287. doi: 10.1038/s41584-019-0202-1 30953036 PMC7097182

[3] Chiu KW, Hash J, Meyers R, Lascelles BDX. The effect of spontaneous osteoarthritis on conditioned pain modulation in the canine model. Sci Rep. 2020 Feb 3;10(1):1694. doi: 10.1038/s41598-020-58499-1 32015421 PMC6997173

[4] Lascelles BDX, Brown DC, Maixner W, Mogil JS. Spontaneous painful disease in companion animals can facilitate the development of chronic pain therapies for humans. Osteoarthritis Cartilage. 2018 Feb;26(2):175–183. doi: 10.1016/j.joca.2017.11.011 Epub 2017 Nov 24. . 29180098

[5] Piel MJ, Kroin JS, van Wijnen AJ, Kc R, Im HJ. Pain assessment in animal models of osteoarthritis. Gene. 2014 Mar 10;537(2):184–8. doi: 10.1016/j.gene.2013.11.091 Epub 2013 Dec 10. 24333346 PMC3950312

[6] Lascelles BDX, Brown DC, Conzemius M, Gill M, Oshinsky ML, Sharkey M. Measurement of chronic pain in companion animals: Priorities for future research and development based on discussions from the Pain in Animals Workshop (PAW) 2017. Vet J. 2019 Oct;252:105370. doi: 10.1016/j.tvjl.2019.105370 Epub 2019 Aug 28. . 31554586

[7] Conzemius MG, Torres BT, Muir P, Evans R, Krotscheck U, Budsberg S. Best practices for measuring and reporting ground reaction forces in dogs. Vet Surg. 2022 Jan 26. doi: 10.1111/vsu.13772 Epub ahead of print. . 35083759

[8] Lee DV. Effects of grade and mass distribution on the mechanics of trotting in dogs. J Exp Biol. 2011 Feb 1;214(Pt 3):402–11. doi: 10.1242/jeb.044487 . 21228199

[9] Tesio L, Rota V. The Motion of Body Center of Mass During Walking: A Review Oriented to Clinical Applications. Front Neurol. 2019 Sep 20;10:999. doi: 10.3389/fneur.2019.00999 31616361 PMC6763727

[10] Baker R. The history of gait analysis before the advent of modern computers. Gait Posture. 2007 Sep; 26(3):331–42. doi: 10.1016/j.gaitpost.2006.10.014 17306979

[11] Erdmann WS. Center of mass of the human body helps in analysis of balance and movement. *MOJ App Bio Biomech*. 2018;2(2):144‒148.

[12] Virmavirta M, Isolehto J: Determining the location of the body׳s center of mass for different groups of physically active people. J Biomech 47:1909–1913, 2014. doi: 10.1016/j.jbiomech.2014.04.001 24742487

[13] Park SJ, Kim CB, Park SC: Anthropometric and biomechanical characteristics on body segments of Koreans. Appl Human Sci 18:91–99, 1999. doi: 10.2114/jpa.18.91 10462840

[14] Damavandi M, Farahpour N, Allard P. Determination of body segment masses and centers of mass using a force plate method in individuals of different morphology. Med Eng Phys 31:1187–1194, 2009. doi: 10.1016/j.medengphy.2009.07.015 19683955

[15] Damavandi M, Dalleau G, Stylianides G, Charles-Hilaire R, Allard P. Head and trunk mass and center of mass position estimations in able-bodied and scoliotic girls. Med Eng Phys 35:1607–1612, 2013. doi: 10.1016/j.medengphy.2013.05.010 23777637

[16] Almeida CW, Castro CH, Pedreira PG, Heymann RE, Szejnfeld VL. Percentage height of center of mass is associated with the risk of falls among elderly women: A case-control study. Gait Posture 34:208–212, 2011. doi: 10.1016/j.gaitpost.2011.04.013 21602047

[17] Adolphe M, Clerval J, Kirchof Z, Lacombe-Delpech R. Center of Mass of Human’s Body Segments, in, Vol 21Mechanics and Mechanical Engineering, 2017, pp 485–497.

[18] Pataky TC, Zatsiorsky VM, Challis JH: A simple method to determine body segment masses in vivo: reliability, accuracy and sensitivity analysis. Clin Biomech (Bristol, Avon) 18:364–368, 2003. 10.1016/s0268-0033(03)00015-9 12689787

[19] Voss K, Galeandro L, Wiestner T, Haessig M, Montavon PM. Relationships of body weight, body size, subject velocity and vertical ground reaction forces in trotting dogs. Vet Surg. 2010; 39:863–869. doi: 10.1111/j.1532-950X.2010.00729.x 20825596

[20] Scott RM, Evans R, Conzemius MG: Efficacy of an oral nutraceutical for the treatment of canine osteoarthritis. A double-blind, randomized, placebo-controlled prospective clinical trial. Vet Comp Orthop Traumatol 30:318–323, 2017. doi: 10.3415/VCOT-17-02-0020 28763523

[21] Mejia S, Duerr FM, Salman M: Comparison of activity levels derived from two accelerometers in dogs with osteoarthritis: Implications for clinical trials. Vet J 252:105355, 2019. doi: 10.1016/j.tvjl.2019.105355 31554587

[22] Eskander BS, Barbar M, Evans RB, Enomoto M, Lascelles BDX, Conzemius MG. Correlation of activity data in normal dogs to distance traveled. Can J Vet Res 84:44–51, 2020. 31920217 PMC6921993

[23] Chambers RD, Yoder NC, Carson AB, Junge C, Allen DE, Prescott LM, et al. Deep Learning Classification of Canine Behavior Using a Single Collar-Mounted Accelerometer: Real-World Validation. Animals (Basel) 11, 2021. doi: 10.3390/ani11061549 34070579 PMC8228965

[24] Tudor-Locke C, Barreira TV, Schuna JM: Comparison of step outputs for waist and wrist accelerometer attachment sites. Med Sci Sports Exerc 47:839–842, 2015. doi: 10.1249/MSS.0000000000000476 25121517

[25] Kamada M, Shiroma EJ, Harris TB, Lee IM. Comparison of physical activity assessed using hip- and wrist-worn accelerometers. Gait Posture 44:23–28, 2016. doi: 10.1016/j.gaitpost.2015.11.005 27004628 PMC4806562

[26] Loprinzi PD, Smith B: Comparison Between Wrist-Worn and Waist-Worn Accelerometry. J Phys Act Health 14:539–545, 2017. doi: 10.1123/jpah.2016-0211 28290761

[27] Lynn R, Pfitzer R, Rogers RR, Ballmann C, Williams TD, Marshall MR. Step-Counting Validity of Wrist-Worn Activity Monitors During Activities With Fixed Upper Extremities. Journal for the Measurement of Physical Behaviour 3:197–203, 2020.

[28] Montoye HJ, Washburn R, Servais S, Ertl A, Webster JG, Nagle FJ. Estimation of energy expenditure by a portable accelerometer. Med Sci Sports Exerc 15:403–407, 1983. 6645869

[29] Matthews CE: Calibration of accelerometer output for adults. Med Sci Sports Exerc 37:S512–522, 2005. doi: 10.1249/01.mss.0000185659.11982.3d 16294114

[30] Matthews CE, Hagströmer M, Pober DM, Bowles HR. Best practices for using physical activity monitors in population-based research. Med Sci Sports Exerc 44:S68–76, 2012. doi: 10.1249/MSS.0b013e3182399e5b 22157777 PMC3543867

[31] Troiano RP, McClain JJ, Brychta RJ, Chen KY. Evolution of accelerometer methods for physical activity research. Br J Sports Med 48:1019–1023, 2014. doi: 10.1136/bjsports-2014-093546 24782483 PMC4141534

[32] Tesio L, Lansade C, Bonnet X, Marvisi N, Facione J, Villa C, et al. Estimation of the body center of mass velocity during gait of people with transfemoral amputation from force plate data integration. Clin Biomech (Bristol, Avon). 2021 Aug;88:105423. 10.1016/j.clinbiomech.2021.105423 34289434

[33] Kobayashi T, Leung AK, Akazawa Y, Hutchins SW. Effect of ankle-foot orthoses on the sagittal plane displacement of the center of mass in patients with stroke hemiplegia: a pilot study. Top Stroke Rehabil. 2012 Jul-Aug;19(4):338–44. doi: 10.1310/tsr1904-338 22750963

