# Supplementary material for: Determination of the center of mass in a heterogeneous population of dogs
Source: PLoS One. 2022 Apr 27;17(4):e0267361. doi: 10.1371/journal.pone.0267361 (PMC9045670; doi:10.1371/journal.pone.0267361)
Supplement: Attachment — Submitted filename: Response to Reviewers.docx [file pone.0267361.s003.docx]

Response to Reviewers

**Determination of the center of mass in a heterogeneous population of dogs**

Reviewers – Thank you for the time you invested into our manuscript. We believe the peer review process has improved the strength of our submission. We have responded to each of your comments below.

Reviewer #1: The manuscript entitled “Determination of the center of mass in a heterogeneous population of dogs” seeks to provide the initial description of the variance associated with static center-of-mass (CoM) measurements in a heterogeneous population of dogs. There are two reported objectives (1. Report on variance of CoM; 2. Describe the relationship between CoM and dog morphology) with a clearly defined and testable hypothesis. Overall, the manuscript is well-written and well-designed. I have only a few comments that should be addressed:

1. In this study, the authors achieve both outlined objectives and describe the results of their hypothesis testing. However, an undefined yet clearly well-integrated objective of this study (discussed throughout the manuscript) is relating the CoM findings to the location of an IMU positioned on the ventral cervical region. Clarification regarding this as an objective within the manuscript is appropriate and could be highly beneficial as its clinical relevance may be more immediately identifiable and significant to readers based on the increased interest and use of IMUs, in the form of activity trackers.

***REVISED. The IMU box was an intentional and important part of our second objective. That objective was modified to read, … and to describe the relationship between CoM, subject morphometrics and an inertial measurement unit (IMU) box positioned ventrally on a neck collar.”***

2. The authors determined CoM in relation to 3 planes (transverse, sagittal, and dorsal) using accepted terminology for the anatomical planes of a standing quadruped. Unfortunately, terminology varies throughout the literature (mostly related to biped vs quadruped differences) and can result in some confusion if not well defined within studies. Additionally, the overwhelming adoption and almost ubiquitous use of human plane terminology (i.e., frontal vs transverse plane) for quadrupedal limbs adds even greater confusion in this area. It is because of these reasons that some form of clarification within the manuscript regarding the exact location of the studied and described planes is warranted. An image/illustration could prove beneficial to readers and would provide immediate clarity.

***REVISED. An illustration has been added as requested.***

3. The authors, at various points throughout the manuscript, touch on the idea that the described CoM measurements are from a static patient and that some variation in CoM location will occur during dynamic motion (the most commonly studied motion in research and clinical endeavors). While this concept is touched on in various areas of the manuscript (ex., lines 222-224), the readers could benefit from a more focus but brief paragraph on this topic within the discussion. Including possible statements on the potential impact of different gaits (asymmetric vs symmetric) on CoM and/or the relevance to IMU measurements and future normalization procedures could be beneficial. Overall, clarifying this key concept may help highlight to readers how these study findings should be interpreted and utilized in the future.

***REVISED. We expanded on this topic so the reader could gain a better understanding of normalization procedures and how they reduce the coefficient of variation to GRF output. In addition, we added a reference that provides great detail with respect to how subject morphometrics can be applied towards normalization of ground reaction forces. Similarly, we expanded on the following paragraph that addresses CoM and IMU data in an effort to explain to the reader how acceleration measured by an IMU is influenced by distance from the CoM (e.g. “…the CoM is the single location where force can induce linear acceleration with angular acceleration). We did not address specific methodology about how to normalize IMU data. We believe that extends beyond this paper and our goal of bringing up these topics is for investigators to at consider that differences in subject CoM could influence their data.***

4. Lines 96, 103, 108 – Please clarify the information after “CoM” that is in parentheses. This may be related to comment #2, but the relationship between the listed terms (i.e. “dorsal to ventral” on line 108) and the defined plane is unclear.

***The revised version includes the previous clarifications in parentheses to assist the reader in anatomical interpretation (rostral to caudal; nose to ischium), (right to left; width of chest), (dorsal to ventral; withers to ventral part of chest). This information along with the additional illustration we hope provides clear anatomical context.***

5. Lines 155-161 – It is unclear if the accompanying value to the mean is the SD. This should be clarified within the text.

***REVISED. This was clarified in the text***.

6. Lines 144-151 – For consistency, it may be beneficial to represent the SD in a similar manner (with our without a “+/-”) to that described lines 155-161 (assuming that those lines represent the SD—see previous comment).

***REVISED. This was clarified in the text***.

Reviewer #2: This manuscript describes research that is an essential step in refining the use of accelerometers to measure activity in dogs. It is a valuable contribution to the literature.

General comments.
1. line 70. Can you say what a reaction board is before describing its construction? I'm not sure readers will immediately understand what a reaction board is. For example, something like, "A reaction board is a triangular platform with one apex that rests on the force plate and is used to support a sedated dog. The reaction board has a known CoG that is accounted for in calculating a dog’s CoG."

***REVISED. This revised manuscript now reads, “A reaction board [12-16] is a platform that has one end rest on a force platform and is used to support the subject. It has a known CoM that is accounted for in calculating the subject’s CoM. The reaction board was made of pinewood and…”***

2. line 94. A dog has four CoG: The 3D CoG and one for each plane. It may help readers if you explicitly say that.

***REVISED. The following sentence was added at the beginning of the paragraph, “The CoM can be defined by each plane (transverse, sagittal and dorsal) and a three-dimensional CoM.”***

3. line 94-95. Is the sentence "The method used did not attempt to identify limb segment CoM." necessary at this point in the paper? It distracted me because I paused to think about what it meant. Scrap it if you don't need it.

***REVISED. The sentence was deleted as recommended.***

4. line 97. Change "board" to "reaction board."

***REVISED. The sentence was revised as recommended.***

5. line 172. the standard deviation of what?

***REVISED. This sentence now reads, “In this population of dogs, the standard deviation of the mean distance from the 3DCoM to the IMU box had an 81% dispersion.”***

6. line 191. This may be the most important paragraph of the paper because it describes how your results can be used. The problem is that I got stuck on the word "normalize" because there is more than one way to normalize variables. The normalization equation doesn't return a percent, such as normalizing bodyweight for GRF. How do we use the normalization equation to normalize? In fact, what are we normalizing?

***REVISED. We expanded on this topic so the reader could gain a better understanding of normalization procedures and how they reduce the coefficient of variation to GRF output. In addition, we added a reference that provides great detail with respect to how subject morphometrics can be applied towards normalization of ground reaction forces. Similarly, we expanded on the following paragraph that addresses CoM and IMU data in an effort to explain to the reader how acceleration measured by an IMU is influenced by distance from the CoM (e.g. “…the CoM is the single location where force can induce linear acceleration with angular acceleration). We did not address specific methodology about how to normalize IMU data. We believe that extends beyond this paper and our goal of bringing up these topics is for investigators to at consider that differences in subject CoM could influence their data.***

7. line 196. Can you give a sentence describing how the equation would be used to reduce variability?
