# Supplementary material for: Determination of the center of mass in a heterogeneous population of dogs
Source: PLoS One. 2022 Apr 27;17(4):e0267361. doi: 10.1371/journal.pone.0267361 (PMC9045670; doi:10.1371/journal.pone.0267361)
Supplement: Peer review history

# Peer review: Decision Letter 0

**DOI:** 10.1371/journal.pone.0267361.r001

**Authors:** Richard Evans

31 Mar 2022

PONE-D-22-07085Determination of the center of mass in a heterogeneous population of dogsPLOS ONE

Dear Dr. Conzemius,

Thank you for submitting your manuscript to PLOS ONE. After careful consideration, we feel that it has merit but does not fully meet PLOS ONE’s publication criteria as it currently stands. Therefore, we invite you to submit a revised version of the manuscript that addresses the points raised during the review process.

The reviewers felt that your manuscript is a strong one and a solid contribution to the literature. One reviewer's recommendations are less focused and somewhat open to interpretation. My recommendation is to do your best to address his or her issues, but the paper is already quite polished, and excessive verbiage should be avoided at this point. Also, I have uploaded a word document with my suggested grammar changes. Please review them and accept or reject them as you see fit.

Please submit your revised manuscript by May 15 2022 11:59PM. If you will need more time than this to complete your revisions, please reply to this message or contact the journal office at plosone@plos.org. When you're ready to submit your revision, log on to https://www.editorialmanager.com/pone/ and select the 'Submissions Needing Revision' folder to locate your manuscript file.

Please include the following items when submitting your revised manuscript:

- A rebuttal letter that responds to each point raised by the academic editor and reviewer(s). You should upload this letter as a separate file labeled 'Response to Reviewers'.
- A marked-up copy of your manuscript that highlights changes made to the original version. You should upload this as a separate file labeled 'Revised Manuscript with Track Changes'.
- An unmarked version of your revised paper without tracked changes. You should upload this as a separate file labeled 'Manuscript'.

If you would like to make changes to your financial disclosure, please include your updated statement in your cover letter. Guidelines for resubmitting your figure files are available below the reviewer comments at the end of this letter.

If applicable, we recommend that you deposit your laboratory protocols in protocols.io to enhance the reproducibility of your results. Protocols.io assigns your protocol its own identifier (DOI) so that it can be cited independently in the future. For instructions see: https://journals.plos.org/plosone/s/submission-guidelines#loc-laboratory-protocols. Additionally, PLOS ONE offers an option for publishing peer-reviewed Lab Protocol articles, which describe protocols hosted on protocols.io. Read more information on sharing protocols at https://plos.org/protocols?utm_medium=editorial-email&utm_source=authorletters&utm_campaign=protocols.

We look forward to receiving your revised manuscript.

Kind regards,

Richard Evans

Academic Editor

PLOS ONE

Journal Requirements:

When submitting your revision, we need you to address these additional requirements.

1. Please ensure that your manuscript meets PLOS ONE's style requirements, including those for file naming. The PLOS ONE style templates can be found at 

https://journals.plos.org/plosone/s/file?id=wjVg/PLOSOne_formatting_sample_main_body.pdf and 

https://journals.plos.org/plosone/s/file?id=ba62/PLOSOne_formatting_sample_title_authors_affiliations.pdf

2. Please review your reference list to ensure that it is complete and correct. If you have cited papers that have been retracted, please include the rationale for doing so in the manuscript text, or remove these references and replace them with relevant current references. Any changes to the reference list should be mentioned in the rebuttal letter that accompanies your revised manuscript. If you need to cite a retracted article, indicate the article’s retracted status in the References list and also include a citation and full reference for the retraction notice.

[Note: HTML markup is below. Please do not edit.]

Reviewers' comments:

Reviewer's Responses to Questions

**Comments to the Author**

1. Is the manuscript technically sound, and do the data support the conclusions?

The manuscript must describe a technically sound piece of scientific research with data that supports the conclusions. Experiments must have been conducted rigorously, with appropriate controls, replication, and sample sizes. The conclusions must be drawn appropriately based on the data presented. 

Reviewer #1: Yes

Reviewer #2: Yes

**********

2. Has the statistical analysis been performed appropriately and rigorously? 

Reviewer #1: Yes

Reviewer #2: Yes

**********

3. Have the authors made all data underlying the findings in their manuscript fully available?

The PLOS Data policy requires authors to make all data underlying the findings described in their manuscript fully available without restriction, with rare exception (please refer to the Data Availability Statement in the manuscript PDF file). The data should be provided as part of the manuscript or its supporting information, or deposited to a public repository. For example, in addition to summary statistics, the data points behind means, medians and variance measures should be available. If there are restrictions on publicly sharing data—e.g. participant privacy or use of data from a third party—those must be specified.

Reviewer #1: Yes

Reviewer #2: Yes

**********

4. Is the manuscript presented in an intelligible fashion and written in standard English?

PLOS ONE does not copyedit accepted manuscripts, so the language in submitted articles must be clear, correct, and unambiguous. Any typographical or grammatical errors should be corrected at revision, so please note any specific errors here.

Reviewer #1: Yes

Reviewer #2: Yes

**********

5. Review Comments to the Author

Please use the space provided to explain your answers to the questions above. You may also include additional comments for the author, including concerns about dual publication, research ethics, or publication ethics. (Please upload your review as an attachment if it exceeds 20,000 characters)

Reviewer #1: The manuscript entitled “Determination of the center of mass in a heterogeneous population of dogs” seeks to provide the initial description of the variance associated with static center-of-mass (CoM) measurements in a heterogeneous population of dogs. There are two reported objectives (1. Report on variance of CoM; 2. Describe the relationship between CoM and dog morphology) with a clearly defined and testable hypothesis. Overall, the manuscript is well-written and well-designed. I have only a few comments that should be addressed:

1. In this study, the authors achieve both outlined objectives and describe the results of their hypothesis testing. However, an undefined yet clearly well-integrated objective of this study (discussed throughout the manuscript) is relating the CoM findings to the location of an IMU positioned on the ventral cervical region. Clarification regarding this as an objective within the manuscript is appropriate and could be highly beneficial as its clinical relevance may be more immediately identifiable and significant to readers based on the increased interest and use of IMUs, in the form of activity trackers.

2. The authors determined CoM in relation to 3 planes (transverse, sagittal, and dorsal) using accepted terminology for the anatomical planes of a standing quadruped. Unfortunately, terminology varies throughout the literature (mostly related to biped vs quadruped differences) and can result in some confusion if not well defined within studies. Additionally, the overwhelming adoption and almost ubiquitous use of human plane terminology (i.e., frontal vs transverse plane) for quadrupedal limbs adds even greater confusion in this area. It is because of these reasons that some form of clarification within the manuscript regarding the exact location of the studied and described planes is warranted. An image/illustration could prove beneficial to readers and would provide immediate clarity.

3. The authors, at various points throughout the manuscript, touch on the idea that the described CoM measurements are from a static patient and that some variation in CoM location will occur during dynamic motion (the most commonly studied motion in research and clinical endeavors). While this concept is touched on in various areas of the manuscript (ex., lines 222-224), the readers could benefit from a more focus but brief paragraph on this topic within the discussion. Including possible statements on the potential impact of different gaits (asymmetric vs symmetric) on CoM and/or the relevance to IMU measurements and future normalization procedures could be beneficial. Overall, clarifying this key concept may help highlight to readers how these study findings should be interpreted and utilized in the future.

4. Lines 96, 103, 108 – Please clarify the information after “CoM” that is in parentheses. This may be related to comment #2, but the relationship between the listed terms (i.e. “dorsal to ventral” on line 108) and the defined plane is unclear.

5. Lines 155-161 – It is unclear if the accompanying value to the mean is the SD. This should be clarified within the text.

6. Lines 144-151 – For consistency, it may be beneficial to represent the SD in a similar manner (with our without a “+/-”) to that described lines 155-161 (assuming that those lines represent the SD—see previous comment).

Reviewer #2: This manuscript describes research that is an essential step in refining the use of accelerometers to measure activity in dogs. It is a valuable contribution to the literature.

General comments.

1. line 70. Can you say what a reaction board is before describing its construction? I'm not sure readers will immediately understand what a reaction board is. For example, something like, "A reaction board is a triangular platform with one apex that rests on the force plate and is used to support a sedated dog. The reaction board has a known CoG that is accounted for in calculating a dog’s CoG."

2. line 94. A dog has four CoG: The 3D CoG and one for each plane. It may help readers if you explicitly say that.

3. line 94-95. Is the sentence "The method used did not attempt to identify limb segment CoM." necessary at this point in the paper? It distracted me because I paused to think about what it meant. Scrap it if you don't need it.

4. line 97. Change "board" to "reaction board."

5. line 172. the standard deviation of what?

6. line 191. This may be the most important paragraph of the paper because it describes how your results can be used. The problem is that I got stuck on the word "normalize" because there is more than one way to normalize variables. The normalization equation doesn't return a percent, such as normalizing bodyweight for GRF. How do we use the normalization equation to normalize? In fact, what are we normalizing?

7. line 196. Can you give a sentence describing how the equation would be used to reduce variability?

**********

6. PLOS authors have the option to publish the peer review history of their article (what does this mean?). If published, this will include your full peer review and any attached files.

If you choose “no”, your identity will remain anonymous but your review may still be made public.

**Do you want your identity to be public for this peer review?** For information about this choice, including consent withdrawal, please see our Privacy Policy.

Reviewer #1: No

Reviewer #2: No

[NOTE: If reviewer comments were submitted as an attachment file, they will be attached to this email and accessible via the submission site. Please log into your account, locate the manuscript record, and check for the action link "View Attachments". If this link does not appear, there are no attachment files.]

While revising your submission, please upload your figure files to the Preflight Analysis and Conversion Engine (PACE) digital diagnostic tool, https://pacev2.apexcovantage.com/. PACE helps ensure that figures meet PLOS requirements. To use PACE, you must first register as a user. Registration is free. Then, login and navigate to the UPLOAD tab, where you will find detailed instructions on how to use the tool. If you encounter any issues or have any questions when using PACE, please email PLOS at figures@plos.org. Please note that Supporting Information files do not need this step.

Attachment Submitted filename: Edited COM Manuscript - PLos ONE.docx Click here for additional data file.

# Author comment: Author response to Decision Letter 0

**DOI:** 10.1371/journal.pone.0267361.r002

1 Apr 2022

Response to Reviewers

Determination of the center of mass in a heterogeneous population of dogs

Reviewers – Thank you for the time you invested into our manuscript. We believe the peer review process has improved the strength of our submission. We have responded to each of your comments below.

Reviewer #1: The manuscript entitled “Determination of the center of mass in a heterogeneous population of dogs” seeks to provide the initial description of the variance associated with static center-of-mass (CoM) measurements in a heterogeneous population of dogs. There are two reported objectives (1. Report on variance of CoM; 2. Describe the relationship between CoM and dog morphology) with a clearly defined and testable hypothesis. Overall, the manuscript is well-written and well-designed. I have only a few comments that should be addressed:

1. In this study, the authors achieve both outlined objectives and describe the results of their hypothesis testing. However, an undefined yet clearly well-integrated objective of this study (discussed throughout the manuscript) is relating the CoM findings to the location of an IMU positioned on the ventral cervical region. Clarification regarding this as an objective within the manuscript is appropriate and could be highly beneficial as its clinical relevance may be more immediately identifiable and significant to readers based on the increased interest and use of IMUs, in the form of activity trackers.

REVISED. The IMU box was an intentional and important part of our second objective. That objective was modified to read, … and to describe the relationship between CoM, subject morphometrics and an inertial measurement unit (IMU) box positioned ventrally on a neck collar.”

2. The authors determined CoM in relation to 3 planes (transverse, sagittal, and dorsal) using accepted terminology for the anatomical planes of a standing quadruped. Unfortunately, terminology varies throughout the literature (mostly related to biped vs quadruped differences) and can result in some confusion if not well defined within studies. Additionally, the overwhelming adoption and almost ubiquitous use of human plane terminology (i.e., frontal vs transverse plane) for quadrupedal limbs adds even greater confusion in this area. It is because of these reasons that some form of clarification within the manuscript regarding the exact location of the studied and described planes is warranted. An image/illustration could prove beneficial to readers and would provide immediate clarity.

REVISED. An illustration has been added as requested.

3. The authors, at various points throughout the manuscript, touch on the idea that the described CoM measurements are from a static patient and that some variation in CoM location will occur during dynamic motion (the most commonly studied motion in research and clinical endeavors). While this concept is touched on in various areas of the manuscript (ex., lines 222-224), the readers could benefit from a more focus but brief paragraph on this topic within the discussion. Including possible statements on the potential impact of different gaits (asymmetric vs symmetric) on CoM and/or the relevance to IMU measurements and future normalization procedures could be beneficial. Overall, clarifying this key concept may help highlight to readers how these study findings should be interpreted and utilized in the future.

REVISED. We expanded on this topic so the reader could gain a better understanding of normalization procedures and how they reduce the coefficient of variation to GRF output. In addition, we added a reference that provides great detail with respect to how subject morphometrics can be applied towards normalization of ground reaction forces. Similarly, we expanded on the following paragraph that addresses CoM and IMU data in an effort to explain to the reader how acceleration measured by an IMU is influenced by distance from the CoM (e.g. “…the CoM is the single location where force can induce linear acceleration with angular acceleration). We did not address specific methodology about how to normalize IMU data. We believe that extends beyond this paper and our goal of bringing up these topics is for investigators to at consider that differences in subject CoM could influence their data.

4. Lines 96, 103, 108 – Please clarify the information after “CoM” that is in parentheses. This may be related to comment #2, but the relationship between the listed terms (i.e. “dorsal to ventral” on line 108) and the defined plane is unclear.

The revised version includes the previous clarifications in parentheses to assist the reader in anatomical interpretation (rostral to caudal; nose to ischium), (right to left; width of chest), (dorsal to ventral; withers to ventral part of chest). This information along with the additional illustration we hope provides clear anatomical context.

5. Lines 155-161 – It is unclear if the accompanying value to the mean is the SD. This should be clarified within the text.

REVISED. This was clarified in the text.

6. Lines 144-151 – For consistency, it may be beneficial to represent the SD in a similar manner (with our without a “+/-”) to that described lines 155-161 (assuming that those lines represent the SD—see previous comment).

REVISED. This was clarified in the text.

Reviewer #2: This manuscript describes research that is an essential step in refining the use of accelerometers to measure activity in dogs. It is a valuable contribution to the literature.

General comments.

1. line 70. Can you say what a reaction board is before describing its construction? I'm not sure readers will immediately understand what a reaction board is. For example, something like, "A reaction board is a triangular platform with one apex that rests on the force plate and is used to support a sedated dog. The reaction board has a known CoG that is accounted for in calculating a dog’s CoG."

REVISED. This revised manuscript now reads, “A reaction board [12-16] is a platform that has one end rest on a force platform and is used to support the subject. It has a known CoM that is accounted for in calculating the subject’s CoM. The reaction board was made of pinewood and…”

2. line 94. A dog has four CoG: The 3D CoG and one for each plane. It may help readers if you explicitly say that.

REVISED. The following sentence was added at the beginning of the paragraph, “The CoM can be defined by each plane (transverse, sagittal and dorsal) and a three-dimensional CoM.”

3. line 94-95. Is the sentence "The method used did not attempt to identify limb segment CoM." necessary at this point in the paper? It distracted me because I paused to think about what it meant. Scrap it if you don't need it.

REVISED. The sentence was deleted as recommended.

4. line 97. Change "board" to "reaction board."

REVISED. The sentence was revised as recommended.

5. line 172. the standard deviation of what?

REVISED. This sentence now reads, “In this population of dogs, the standard deviation of the mean distance from the 3DCoM to the IMU box had an 81% dispersion.”

6. line 191. This may be the most important paragraph of the paper because it describes how your results can be used. The problem is that I got stuck on the word "normalize" because there is more than one way to normalize variables. The normalization equation doesn't return a percent, such as normalizing bodyweight for GRF. How do we use the normalization equation to normalize? In fact, what are we normalizing?

REVISED. We expanded on this topic so the reader could gain a better understanding of normalization procedures and how they reduce the coefficient of variation to GRF output. In addition, we added a reference that provides great detail with respect to how subject morphometrics can be applied towards normalization of ground reaction forces. Similarly, we expanded on the following paragraph that addresses CoM and IMU data in an effort to explain to the reader how acceleration measured by an IMU is influenced by distance from the CoM (e.g. “…the CoM is the single location where force can induce linear acceleration with angular acceleration). We did not address specific methodology about how to normalize IMU data. We believe that extends beyond this paper and our goal of bringing up these topics is for investigators to at consider that differences in subject CoM could influence their data.

7. line 196. Can you give a sentence describing how the equation would be used to reduce variability?

Attachment Submitted filename: Response to Reviewers.docx Click here for additional data file.

# Editor report: Decision Letter 1

**DOI:** 10.1371/journal.pone.0267361.r003

**Authors:** Richard Evans

7 Apr 2022

Determination of the center of mass in a heterogeneous population of dogs

PONE-D-22-07085R1

Dear Dr. Conzemius,

We’re pleased to inform you that your manuscript has been judged scientifically suitable for publication and will be formally accepted for publication once it meets all outstanding technical requirements.

Within one week, you’ll receive an e-mail detailing the required amendments. When these have been addressed, you’ll receive a formal acceptance letter and your manuscript will be scheduled for publication.

An invoice for payment will follow shortly after the formal acceptance. To ensure an efficient process, please log into Editorial Manager at http://www.editorialmanager.com/pone/, click the 'Update My Information' link at the top of the page, and double check that your user information is up-to-date. If you have any billing related questions, please contact our Author Billing department directly at authorbilling@plos.org.

If your institution or institutions have a press office, please notify them about your upcoming paper to help maximize its impact. If they’ll be preparing press materials, please inform our press team as soon as possible -- no later than 48 hours after receiving the formal acceptance. Your manuscript will remain under strict press embargo until 2 pm Eastern Time on the date of publication. For more information, please contact onepress@plos.org.

Kind regards,

Richard Evans

Academic Editor

PLOS ONE

Additional Editor Comments:

Thank you for addressing the reviewers' comments thoroughly, carefully and in detail. 

# Editor report: Acceptance letter

**DOI:** 10.1371/journal.pone.0267361.r004

**Authors:** Richard Evans

18 Apr 2022

PONE-D-22-07085R1 

Determination of the center of mass in a heterogeneous population of dogs 

Dear Dr. Conzemius:

I'm pleased to inform you that your manuscript has been deemed suitable for publication in PLOS ONE. Congratulations! Your manuscript is now with our production department. 

If your institution or institutions have a press office, please let them know about your upcoming paper now to help maximize its impact. If they'll be preparing press materials, please inform our press team within the next 48 hours. Your manuscript will remain under strict press embargo until 2 pm Eastern Time on the date of publication. For more information please contact onepress@plos.org.

If we can help with anything else, please email us at plosone@plos.org. 

Thank you for submitting your work to PLOS ONE and supporting open access. 

Kind regards, 

PLOS ONE Editorial Office Staff

on behalf of

Dr. Richard Evans 

Academic Editor

PLOS ONE
